# Random Forest for Automatic Feature Importance Estimation and Selection for Explainable Postural Stability of a Multi-Factor Clinical Test

**DOI:** 10.3390/s21175930

**Published:** 2021-09-03

**Authors:** Tomas Mendoza, Chia-Hsuan Lee, Chien-Hua Huang, Tien-Lung Sun

**Affiliations:** 1 Department of Industrial Engineering and Management, Yuan Ze University, 135 Yuan Tung Road, Chungli District, Taoyuan 320, Taiwan; s1078907@mail.yzu.edu.tw; 2 Department of Industrial Management, National Taiwan University of Science and Technology, No. 43, Sec. 4, Keelung Road, Da’an District, Taipei 106, Taiwan; sweat0430@mail.ntust.edu.tw; 3 Department of Eldercare, Central Taiwan University of Science and Technology, Taipei 106, Taiwan; 108184@ctust.edu.tw

**Keywords:** multiscale entropy, permutation entropy, timed up and go (TUG), inertial sensor, short form berg balance scale (SFBBS), features, random forest, fall risk, community-dwelling elderly

## Abstract

Falling is a common incident that affects the health of elder adults worldwide. Postural instability is one of the major contributors to this problem. In this study, we propose a supplementary method for measuring postural stability that reduces doctor intervention. We used simple clinical tests, including the timed-up and go test (TUG), short form berg balance scale (SFBBS), and short portable mental status questionnaire (SPMSQ) to measure different factors related to postural stability that have been found to increase the risk of falling. We attached an inertial sensor to the lower back of a group of elderly subjects while they performed the TUG test, providing us with a tri-axial acceleration signal, which we used to extract a set of features, including multi-scale entropy (MSE), permutation entropy (PE), and statistical features. Using the score for each clinical test, we classified our participants into fallers or non-fallers in order to (1) compare the features calculated from the inertial sensor data, and (2) compare the screening capabilities of the multifactor clinical test against each individual test. We use random forest to select features and classify subjects across all scenarios. The results show that the combination of MSE and statistic features overall provide the best classification results. Meanwhile, PE is not an important feature in any scenario in our study. In addition, a *t*-test shows that the multifactor test of TUG and BBS is a better classifier of subjects in this study.

## 1. Introduction

Almost 30% of adults over 65 years old worldwide experience falls [1,2], and falls represent the main cause of their injuries, which include movement impairment, fractures, long-term or permanent disabilities, and death [3]. Suffering from a fall represents not only a great health risk, but is also associated with important economic costs which can range from hospitalizations to long-term home care [4]. Furthermore, adults who have suffered from a traumatic fall can be affected by the fear of suffering from a second fall (FOF), which increases the probability of experiencing recurrent episodes [5]. Approximately 50% of adults who have suffered from a fall are considered potential recurrent fallers [6].

Falls are a multifactorial problem that can mainly be attributed to intrinsic (behavioral, physical, and cognitive) and environmental reasons [7]. Among the intrinsic factors, poor balance and gait abnormalities are estimated to be related to 10–25% of falls [8]. Moreover, previous studies have proven that suffering problems with balance [9,10,11,12] and mobility [5,13,14,15,16] can greatly increase the probabilities of experiencing a fall. The abundant health implications associated with falling has fostered a substantial number of studies that focus on fall risk assessment, as it will become essential in the creation of specialized healthcare programs for aging groups [17].

A key aspect of fall risk assessment is detecting persons at risk to prevent occurrences and the consequent medical complications that can affect health. Common clinical tools used by medical professionals to assess fall risk include questionnaires, gait analysis, and physical tests [18]. Unfortunately, continuous monitoring of fall-related factors such as mobility or balance is a challenging task, as professional resources such as physiotherapists, nurses, or doctors are limited when compared to the size of the population at risk of falling. In addition, any prolonged use of these tools represents elevated economic costs to hospitals, healthcare systems, and patients. Nevertheless, these drawbacks present an opportunity for the implementation of inertial sensors, which are a more commercially available, non-intrusive, lightweight, and affordable means of facilitating the assessment of mobility and balance. Additionally, data from inertial sensors has the sensitivity required to be used for clinical studies [19], for example, assisting doctors in screening fall-risk subjects [20].

In order to obtain valuable information using inertial sensors, subjects must perform tasks that can allow researchers to study factors such as mobility or balance which are associated with falls. Recent studies have focused on analyzing gait-related features extracted from the inertial sensor data of subjects walking or performing a clinical test in order to predict falls. Over a period of 12 months, Wang et al. [21] extracted step rate and movement vigor from subjects walking on stairs and a flat surface to estimate their risk of falling. Ponti et al. [22] combined gait features with statistical features to screen a group of 41 community-dwelling elderly patients into fallers and non-fallers. Howcroft et al. [23] used a set of inertial and pressure sensors located in different parts of the subjects’ bodies in order to calculate gait and statistic features from the data collected from participants performing single (walking) and dual tasks (walking with an additional cognitive load). An important shortcoming of these studies is the fact that using gait-related features requires further processing, segmentation, or signal analysis. These generally need to be conducted prior to estimating fall risk, a time-consuming procedure that further complicates analysis. A recent study focuses on using statistical features calculated from the TUG acceleration data from a group of stroke patients in order to classify them into fallers or non-fallers [24]. However, they segmented the TUG inertial sensor data prior to calculating the statistical features, which requires special knowledge and extensive analysis of the signals captured by the sensor, and represents an obstacle when calculating real-world situations where resources are limited. Therefore, our study aims to address this problem as the method proposed focuses on calculating features from the inertial sensor data automatically, no processing required.

With the importance that Deep Learning (DL) has gathered within the research community, recent studies have proven that DL can be used for making meaningful and explainable predictions on medical time series data [25,26]. However, while ConvNets can be used to extract features from inertial sensor data for fall risk classification [27,28,29,30], prior to the feature extraction, the CNN algorithm needs large amounts of data to be trained. This can be challenging to obtain in a healthcare-related scenario, where the availability of medical personnel and willingness of subjects to participate are limited. Moreover, calculating spatio-temporal features using a CNN would require manual labeling of data, which is a time-consuming process. Finally, studies that use ConvNets to calculate features to classify subjects into fall risk fail to discuss feature importance. Our proposed method addresses these aspects by requiring no training to calculate the features, using simple algorithms (such as peak detection) to automatically calculate the spatio-temporal features, and presenting the reader with a set of features that can be used to classify subjects with fall risk.

The TUG acceleration signal measured by an inertial sensor is highly complex, which makes screening fallers challenging. Computing the complexity of a signal provides useful information for the analysis of physical time series [31], such as the gait signal captured by our sensor. Multiscale Entropy (MSE) is a variation of traditional entropy, which facilitates the quantization of complexity of physical and physiological time series. MSE has been implemented to assess differences in balance by evaluating the complexity between different groups of subjects [32]. Costa [33] analyzed the complexity of the gait signal of subjects while performing a free or paced walk, and concluded that MSE was capable of detecting characteristics of the signal that other statistic tools could not. Riva et al. [34] found MSE to have a positive relationship with fall history, making it a useful instrument to identify individuals at risk of falling. In a similar study, Lee et al. [35] concluded that MSE can be used as a tool to screen falling behavior among elder adults in a community-dwelling setting. The computation of MSE, however, requires the calculation of multiple time series under different scales, which can be time consuming, making it difficult to use when performing immediate decisions. Therefore, Band and Pompe [36] proposed the use of Permutation Entropy (PE), as it is computationally simple, making it ideal for larger time series or databases. The implementation of PE for gait analysis was studied by Lee et al. [37], where an inertial sensor was used to measure the gait information from a subject performing the TUG test. Subjects also performed a short-form berg balance scale test (SFBBS) as a means to measure balance. They used a set of statistical, PE, and weighted permutation entropy (WPE) features to successfully estimate the SFBBS score, which can provide doctors with information on the fall risk of patients. Despite promising results, this study failed to implement a multifactorial assessment test which has been proven to be more effective than a single clinical tool at capturing the complex nature of falls [38,39]. Furthermore, the study did not compare the PE and MSE, as both tools were designed to measure the complexity of a signal. This encouraged us to compare the importance that MSE and PE features can have in the analysis of gait signals while predicting scores for multiple clinical tests.

Our research focuses on studying the application of combined inertial sensors with multifactor assessments, namely (i) the Timed-up and Go test, (ii) Short-Form Berg Balance Scale, and (iii) Short Portable Mental Status Questionnaire to develop an auxiliary tool for medical professionals to assess mobility and balance. The main highlights of this research are: first, we use features that can be automatically extracted or calculated from data collected by inertial sensors without any processing or segmentation, thereby reducing the burden on medical staff and creating a tool that provides data that can be easily interpreted by the doctors or physicians across hospitals. Second, we also compare the performance of our method across different clinical tests in order to increase the robustness of our model by estimating multiple factors that can cause falls among the elder population. Finally, we use MSE features as a means to measure the complexity of the TUG signals and compare their impact to the classification performance against permutation entropy. 

## 2. Materials and Methods

### 2.1. General Approach

Wearing an inertial sensor capable of measuring acceleration in three directions, subjects performed a series of balance and mobility tests. With the data collected by the sensor, we calculated a set of features, which included statistic, MSE, and PE. We used these features to train a Random Forest classifier in order to predict the subject’s scores in the multifactor assessment. From these results, we estimated feature importance and compared the model performance when using the most important features for each clinical test. By doing this, we were able to determine a set of features that can best predict the mobility and stability scores of the participants in our study. 

### 2.2. Subjects

Assisted by a team of medical professionals, which included physiotherapists, functional therapists, and rehabilitation physicians, we performed a series of clinical tests between April 2014 and May 2015 in a hospital in central Taiwan to assess fall risk among the elderly population. Subjects who participated in the study wore a belt around their waist with a tri-axial inertial sensor attached to it, which was located at their lower back while they performed a series of tests (which we discuss in more detail in Section 2.3). At the end of the study, we collected inertial acceleration data from 65 different elderly adults (average age 76.12 ± 6.99 years). The recruitment criteria for the participants stated that they must not have previously suffered from any musculoskeletal injuries, they must not have any history of central nervous system injuries, and they had to be able to walk independently in order to perform the clinical tests. Despite being collected almost seven years ago, this dataset remains relevant as it is focused on a problem that continues to affect the health of a growing elderly population. Moreover, as it was collected using a sensitive sensor, following a careful and scientific methodology from a wide range of elder subjects, and with the support and supervision of professional medical staff, it has allowed our team to continue to develop different methodologies to study it. A summary of the demographic data from the subjects who participated in the study is included in Table 1. This study was approved on 19 May 2015 by the Institutional Review Board of Tsaotun Psychiatric Center, Ministry of Health and Welfare’s research ethics board (approval number 104013). 

### 2.3. Clinical Tests

Due to the demography of our subject population, we implemented quick and simple clinical assessment tests to study mobility and balance, which the participants performed under the supervision of our medical team. This set of tests included:

- The Timed Up and Go test (TUG) [40] is a common clinical test of gait and mobility. Different geriatric institutions recommend its implementation for fall-risk screening [41]. Previous studies have determined the effectiveness of using an inertial sensor during a TUG test to measure mobility [42], as well as to detect frailty [43] which could potentially result in a fall. It has also been proven to be an accurate measurement tool for predicting falls among community-dwelling elder adults [44]. Physicians commonly employ this clinical test in community settings due to its ease of implementation. Before starting the TUG test, subjects sit on a chair in a comfortable position, facing an object on a floor, which is located 3 m in front of them. When the test starts, subjects are asked to stand up, walk naturally towards the object, then return to the chair at their natural pace and sit down. The total time the subjects require to perform this test is recorded and used to label the subjects that performed the test in over 12.47 s as having mobility problems [44]. A summary of the label distribution for each clinical test can be observed in Table 2.
- The Short-Form Berg Balance Scale (SFBBS) [45] is the simplified version of the Berg Balance Scale (BBS) [46] which is used to assess balance. It is easier to perform as it has half the number of activities, greatly reducing the time required to assess a subject. These activities include (i) bending your back forward with outstretched arms, (ii) standing with both feet while keeping eyes closed, (iii) standing with one foot in front of the other, (iv) turning the back and neck to look backwards without moving the feet or knees, (v) bending down to pick up an object from the floor, (vi) standing on one foot while having the other foot in the air, and (vii) standing up from a chair and sitting down again. While subjects perform a SFBBS test, a medical expert evaluates their performance by assigning scores to each activity. The performance criteria states that a score between zero points (subject was unable to perform the activity) and four points (subject completed the activity without problems) is assigned based on the expert’s observations. Therefore, in this study, subjects who scored 28 points were considered to have correct balance since they were able to perform all seven tasks without problems. Meanwhile, subjects with a score below 23 were labeled as having balance problems [47,48].

The Short Portable Mental Status Questionnaire (SPMSQ) [49] is as a sensitive clinical tool to detect brain syndromes such as dementia in elderly adults [50]. Elderly patients diagnosed with dementia have two to three times higher risk of suffering from a fall when compared to patients with healthy cognition [51,52,53,54,55], which makes detecting dementia an important step towards fall risk assessment. A previous study included SPMSQ among the clinical tools used to assess fall-risk factors in a community-dwelling environment [56]. The SPMSQ consists of a set of 10 questions which patients can choose to answer independently or with the help of their family members at home. These questions focus on evaluating cognitive functions such as memory, attention, thinking process, consciousness, general knowledge, and orientation. This provides a preliminary insight on the mental health status of elder adults. Granger et al. [57] determined that a score in the SMPSQ of 60 provides crucial information as patients are transitioning from assisted independence to dependence. In addition, subjects are considered to be at risk of suffering from dementia if they answer three or more questions incorrectly. We used this criterion to label subjects as not having normal brain functionality. 

### 2.4. Wearable Accelerometer

**Figure 1:**
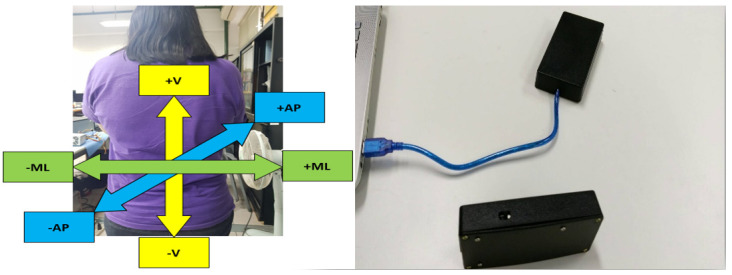
Schematic diagram of the sensor with a visualization of the three axes.

We collected the TUG accelerometer data using a wireless tri-axial accelerometer system (comprised of the Freescale RD3152MMA7260Q accelerometer, a Bluetooth transmitter, a battery as the power source, and an Arduino as the data-processing device). While the subjects performed their clinical tests, the sensor was located at the lower back of the subjects, around the area between the L3 to L5 vertebrae, since previous studies have concluded that this location approximates the center of mass of the human body [58], making this the most common location for similar studies published within the last two decades [59]. This sensor recorded TUG acceleration data in the mediolateral (ML), vertical (V), and anterior–posterior (AP) directions. An illustration of the sensor system with an overview of the axis directions is included in Figure 1. 

### 2.5. Data Analysis

We used Python to automatically calculate the set of features, perform the analysis, and train the classification model. Table 3 summarizes the set of features we used in this study, which we calculated from the unprocessed TUG acceleration signal captured by the inertial sensor. We divided them into statistic, MSE, and PE groups. We selected these features as calculating them requires no signal processing or analysis. Among the statistic set, we calculated the mean, standard deviation, maximum value, minimum value, and zero-crossing rate (ZCR) for each axis. Lee and Sun [60] included ZCR among their feature set, which they used in order to screen fallers from non-fallers. In our study, ZCR measures the frequency at which the gait signal crosses through zero acceleration. The MSE feature set included the average and standard deviation across all time scales and the complexity index. 

#### 2.5.1. MSE Calculation

**Figure 2:**
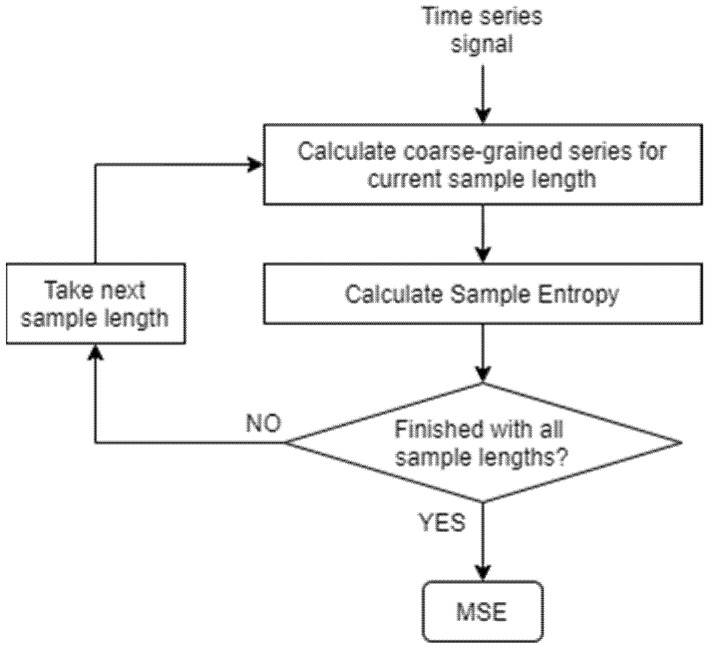
Steps required for calculating MSE from a given time series.

MSE is an effective tool to measure a physiologic time series’ complexity [61]. Costa et al. [33] found that MSE is capable of detecting differences in gait of subjects under pathologic conditions. In our study, we calculated MSE for the entire TUG signal in order to obtain information of the gait of our subjects. A flowchart detailing the steps needed to calculate MSE is illustrated in Figure 2. 

As part of the first step, multiple overlapping segment windows with a length equal to the current scale factor are extracted from a given time series. For each window, the average value for all the data points is calculated, creating a new time series known as coarse-grained time series. The formula used to calculate this is shown in Equation (1), where τ is the scale factor, N is the length of the original time series, and $x_{i}$ is a single data point from the original time series.

(1) $$y_{j}^{\left(\tau \right)}=\frac{1}{\tau }\overset{j\tau }{\underset{i=\left(j-1\right)\tau +1}{\sum }}x_{i};1\leq j\leq \frac{N}{\tau }$$

The next step involves calculating sample entropy (SampEn) for each coarse-grained series. SampEn was developed in order to analyze the complexity of biological time series [62]. SampEn is defined as the negative logarithmic probability of a series having two sets of consecutive data points (of size m+1) with distance < r, given that the same series contains two sets of consecutive data points (of size m) with distance < r. This is expressed in mathematical notation in Equation (2), where N represents the input time series. For our study, we defined these parameters as m = 2 and = 0.15.

(2) $$SampEn\left(N,m,r\right)=-log\frac{d\left[X_{m+1}\left(i\right),X_{m+1}\left(j\right)\right]<r}{d\left[X_{m}\left(i\right),X_{m}\left(j\right)\right]<r}$$

Finally, Costa et al. [61] proposed the use of the complexity index to evaluate fall behavior, which is defined as the summation of the sample entropy values for all scale factors τ. This measurement has been proven to be an effective tool to screen community-dwelling elderly people for falling behavior [35]. The formula used to calculate CI can be observed in Equation (3), where τ represents the scale factor. In our study, we defined τ=10.

(3) $$\overset{n}{\underset{\tau =1}{\sum }}SampEn\left(\tau \right)$$

#### 2.5.2. Permutation Entropy Calculation

PE quantifies complexity by estimating the frequency of sequence patterns within a time series. In order to achieve this, at the first step, it converts a one-dimensional time series into a T−(D−1)τ matrix, where D represents the embedding dimension which defines the size of each column vector in the matrix, and τ represents the embedding time delay which determines the number of time periods that separate the elements of every consecutive pair of columns in the matrix. 

The next step involves converting a one-dimensional time series into a T−(D−1)τ matrix, where D represents the embedding dimension which defines the size of each column vector in the matrix, and τ represents the embedding time delay, which determines the number of time periods that separate the elements of every consecutive pair of columns in our matrix.

As part of the third step, every column in the matrix is mapped into D! unique permutations. These permutations are then sorted in ascending order, which allows the user to obtain the ordinal rankings of the data and their corresponding ordinal patterns. These ordinal patterns are labeled as $\pi_{i},=\left\{r_{0},r_{1},r_{2},\ldots ,r_{D!-1},\right\}$.

Using the ordinal patterns $\pi_{i}$ for each permutation, their relative frequency (defined as the number of times such permutation is present in the time series divided by the total number of sequences) is calculated. This result can be interpreted as the probability of finding each permutation in the time series $p_{i}$.

Finally, using the previously calculated probabilities, the PE value can be calculated following Equation (4). As pointed out by [63], a more regular time series is characterized by having a lower PE value.

(4) $$PE_{D}=\overset{D!}{\underset{i=1}{\sum }}p_{i}log_{2}p_{i}$$

### 2.6. Random Forest for Feature Importance and Classification

In our study, we trained a Random Forest [64] classifier to estimate feature importance. Random Forest for feature selection has been used in problems such as power generation forecasting [65], network intrusion detection [66], and leukemia and cervical cancer classification [67]. To reduce the bias of our model towards the samples in the training set, we employed a 50-fold cross-validation approach. We repeated this process for each clinical test in order to determine the relationship between features to clinical tests. We estimated the importance of each feature by calculating the mean coefficient value for every feature across folds. Once the feature importance for all features was estimated, we proceeded to compare the classification performance of our model by re-training it using the top 5, top 10, and top 15 features. A similar approach was employed in previous research [68] where the authors calculated the mean coefficient scores for each feature, then selected the top 30, 20, 10, and 5 features to test their Random Forest model’s performance. We repeated the 50-fold cross-validation approach to obtain a mean AUC score for each scenario. 

## 3. Results and Discussion

We begin our analysis by classifying every subject with either fall risk or non-fall risk using the scores and the special criteria for each clinical test. Next, we calculated the features from the TUG acceleration data collected by the inertial sensor. Using Python’s library *Scikit-Learn* [69], we estimated feature importance with a Random Forest (RF) classifier, and compared it across clinical tests. We re-trained the RF classifier using the top 5, 10, and 15 features and tested the model’s performance for each clinical test. We proceeded to compare the impact of including and excluding MSE from the feature set as to estimate its effect in measuring balance and mobility, two key factors in fall-risk classification. Finally, we compare the model’s performance across the multifactor clinical tests, in order to determine which assessment tool has the best screening capabilities for fall risk among the community-dwelling elderly subjects who participated in our study. The discussion of our results is divided into three main segments: (a) feature selection for each clinical test, (b) classification performance for each clinical test under multiple criteria for feature selection, and (c) comparison between the classification performance with and without MSE features. 

### 3.1. Feature Selection for Each Clinical Test

The top 5, 10, and 15 features that our model determined to be the most important for each clinical test are summarized in Table 4. The results show that Standard Deviation (for all three directions), Maximum Value (for ML, and V directions), Minimum Value (V), Zero-Crossing Rate (ML), and MSE Mean (ML) are present within these features for all clinical tests. This is consistent with [37], as the author found multiple of these features to have an impact on the screening performance of the model. Additionally, these results indicate that from an axis point of view, ML and V are critical in the classification of the subjects. Furthermore, the selection of MSE features as important for the screening of fall-risk subjects is also consistent with previous studies [35], indicating that the measurement of signal complexity can help to detect differences in balance and mobility. The remaining features are different for each case since every clinical test measures different characteristics of the subject’s posture. Additionally, PE was selected to be among the top features for two clinical tests; however, its importance is clearly lower than MSE. We attribute this to PE’s focus on sample order without considering amplitude. Moreover, having multiple parameters pairs can also lead to testing problems since the values of PE are directly dependent to the parameter setting, as was discovered by [70].

### 3.2. Classification Performance for Each Clinical Test under Multiple Criteria for Feature Selection

After training Random Forest with 50-fold cross validation for multiple criteria of feature selection, we tested each model’s performance by analyzing the mean AUC scores summarized in Table 5. As is evident from the results, the model can classify the subjects according to their respective clinical test scores with high accuracy in most cases. This indicates that the set of features we calculated from the TUG signal are sensitive enough to be used in our study, which is consistent with the findings of [20] who concluded that inertial sensors can be used in fall-risk assessment studies. From the results, it can also be observed that combining SPMSQ with other clinical tests yields the worst performance as it results in the lowest AUC scores. We attribute this to the nature of the SPMSQ, where the score is based on the answers of a written questionnaire, which are highly subjective. Moreover, this test does not measure any balance or motion from the subject, making it more difficult for the set of features we calculated from the TUG data to estimate its score. In addition, PE features were only present on SPMSQ tests, which clearly indicate that other features such as MSE have a higher importance for the clinical tests that directly test mobility and balance. This table also shows that the best results are obtained when selecting the top five features. Considering that such groups include MSE features, we tested the impact to the model’s performance when removing MSE, and discuss the results in Section 3.3 Classification performance with and without MSE.

In such a comparison, we removed SPMSQ and its combinations as it has the worst screening results, as previously discussed. 

### 3.3. Classification Performance with and without MSE

Table 6 summarizes the comparison in mean AUC scores of the model when MSE is excluded from the feature set. The results summarized in this table show an overall reduction in the model’s performance, emphasizing the importance of MSE to analyze the complex TUG acceleration signal. The importance of MSE in our model goes in accordance with [34], where it was concluded that MSE can help to identify subjects in risk of suffering from a fall. It can also be observed that including MSE will improve the classification accuracy of the model across clinical tests, independent of the percentage of features selected. In addition, the multifactor test outperforms the single BBS assessment in all scenarios, which is consistent with previous studies which determined that a multifactor test is better at capturing the complex nature of falls [38,39]. Despite TUG having a higher AUC score than the multifactor test, it is important to point out that the latter is simultaneously assessing both mobility and balance, which are two of the main factors that affect falls. 

A similar tendency is observed on Table 7, where the average precision and average recall values for the classifiers clearly decrease after removing MSE from the feature set. It can also be observed that the highest precision and recall values can be found when using the TOP 10 and TOP 5 features on the multifactor test. This further indicates the importance of using a multifactor test. In addition, the values presented on such table indicate the models are robust and have high classification accuracy.

We also tested the normality of our results from Table 6 using the *Kolgomorov–Smirnov test*, and compiled the results on Table 8. The results of each condition’s *p*-value are > 0.05, showing that the data follows a normal distribution. 

Finally, in Table 9 we included the results of our t-test, which we performed in order to determine whether including or excluding MSE has any statistical impact in the accuracy of each scenario. We found that the “Top 10” and “Top 5” combinations of features have significant statistical differences. Looking back at 4, we can find that the features after T10 indeed do not appear in each combination. Especially after comparing the results in Table 6, it can be found that among the top 10 features, MSE has relative discriminative power. The results from our statistical analysis are consistent with [29]. Additionally, in the t-test we also included the different clinical tools we used for our study. From the results, we can also observe a statistical difference, further highlighting the discrimination capability of MSE. 

## 4. Conclusions

This study analyzed the application of statistical, MSE, and PE features calculated from the inertial sensor data of elder subjects to estimate their scores from multiple clinical tests, as these tests can support medical professionals to screen elder adults for fall risk. We proved that using automatically extracted features from inertial sensor data can provide good screening performance as our model was capable of estimating the multifactor score of the different tests with high accuracy. By analyzing the feature selection, we found that the important features belonged to a combination of statistic and MSE features, indicating that PE was less important when predicting clinical scores. Furthermore, MSE features were present among the top features for all clinical tests. This led to a comparison of the impact in classification AUC score when including and excluding MSE from the set of features to be used in the model. The results for such a test showed that including MSE features increases the performance of the model when estimating BBS, TUG, and TUG + BBS medical scores. We also found the utilization of a multifactor assessment to not only provide better results than the single BBS clinical tool, but also categorize subjects based on mobility and stability, two factors that have been found to be related to falls. In the future, we plan to compare the impact that MSE has in fall risk assessment when two different group of subjects participate in the study. Furthermore, we plan to investigate if the same set of features are selected as important when different sensors are used to collect the data.

## Figures and Tables

**Table 1:** Demographic data of the subjects who participated in this study.

		Number	Mean ± STD
Gender	Female	49	77 ± 6.60
	Male	16	73 ± 6.00
Age	65–70	12	69 ± 1.72
	71–75	25	73 ± 1.53
	76–80	12	78 ± 1.48
	80+	16	85 ± 4.60

**Table 2:** Label distribution of all subjects for each clinical test.

	BBS	TUG	TUG + BBS	SPMSQ	TUG + SPMSQ	BBS + SPMSQ
Healthy	61	57	64	68	69	70
Fall Risk	13	17	10	6	5	4

**Table 3:** List of features extracted from the inertial sensor data.

Feature Type	Feature Name	Direction
Statistic Features	Mean (MEAN) (1–3)	ML,V,AP
	Standard deviation (STD) (4–6)	ML,V,AP
	Maximum value (MAX) (7–9)	ML,V,AP
	Minimum value (MIN) (10–12)	ML,V,AP
	Zero-crossing rate (ZCR) (13–15)	ML,V,AP
Multi Scale Entropy Features	Mean (MSEM) (16–18)	ML,V,AP
	Standard Deviation (MSTD) (19–21)	ML,V,AP
	Complexity Index (CI) (22–24)	ML,V,AP
Permutation Entropy Features	Entropy (PE) (25–27)	ML,V,AP

**Table 4:** Feature importance selection results, with the average feature importance coefficient in parenthesis.

Top Features	BBS	TUG	SPMSQ	TUG + BBS	TUG + SPMSQ	BBS + SPMSQ
	STD (AP) (0.087)	MAX (V) (0.098)	STD (V) (0.078)	CI (ML) (0.079)	STD (V) (0.099)	STD (AP) (0.086)
	ZCR (ML) (0.078)	STD (ML) (0.096)	STD (AP) (0.074)	MSEM (ML) (0.078)	MAX (V) (0.067)	STD (V) (0.081)
	MIN (V) (0.071)	CI (ML) (0.093)	MAX (ML) (0.061)	ZCR (ML) (0.076)	MAX (ML) (065)	MAX (ML) (0.077)
	MSEM (ML) (0.062)	MSEM (ML) (0.085)	STD (ML) (0.049)	STD (AP) (0.068)	MIN (V) (0.063)	MIN (V) (0.067)
TOP 5	CI (ML) (0.060)	STD (V) (0.071)	MAX (V) (0.045)	MAX (V) (0.067)	STD (ML) (0.062)	ZCR (ML) (0.063)
	STD (V) (0.058)	MIN (AP) (0.05)	MIN (V) (0.045)	STD (V) (0.065)	CI (ML) (0.061)	PE (AP) (0.062)
	MAX (V) (0.052)	ZCR (ML) (0.048)	MSEM (AP) (0.044)	MAX (ML) (0.063)	MSEM (ML) (0.054)	STD (ML) (0.053)
	STD (ML) (0.048)	MAX (ML) (0.046)	CI (AP) (0.043)	STD (ML) (0.044)	STD (AP) (0.050)	CI (ML) (0.045)
	MSTD (AP) (0.044)	MIN (V) (0.039)	MAX (AP) (0.043)	MIN (AP) (0.040)	PE (ML) (0.046)	MAX (V) (0.041)
TOP 10	MAX (ML) (0.040)	MIN (ML) (0.037)	MSEM (ML) (0.043)	MIN (V) (0.038)	ZCR (ML) (0.042)	MSEM (ML) (0.038)
	MEAN (V) (0.034)	STD (AP) (0.035)	CI (ML) (0.040)	CI (V) (0.034)	ZCR (AP) (0.040)	MAX (AP) (0.035)
	CI (V) (0.034)	CI (V) (0.027)	MSTD (ML) (0.036)	MSEM (V) (0.034)	MAX (AP) (0.035)	MEAN (V) (0.035)
	MIN (AP) (0.034)	MSEM (AP) (0.025)	MSTD (AP) (0.035)	MAX (AP) (0.033)	PE (AP) (0.031)	CI (AP) (0.034)
	MSEM (V) (0.03)	MSEM (V) (0.024)	ZCR (AP) (0.034)	CI (AP) (0.030)	MIN (AP) (0.029)	MEAN (ML) (0.028)
TOP 15	MEAN (ML) (0.029)	CI (AP) (0.024)	ZCR (ML) (0.033)	MSEM (AP) (0.029)	MSTD (ML) (0.026)	ZCR (V) (0.028)

**Table 5:** RF mean AUC scores for each clinical test.

Clinical Test	Top 15 Features	Top 10 Features	Top 5 Features
BBS	0.828	0.856	0.866
TUG	0.899	0.913	0.921
TUG + BBS	0.883	0.910	0.922
SPMSQ	0.722	0.747	0.680
TUG + SPMSQ	0.814	0.808	0.783
BBS + SPMSQ	0.781	0.801	0.774

**Table 6:** Average AUC classification scores with and without MSE.

	With MSE Features			Without MSE Features		
Clinical Test	Top 15	Top 10	Top 5	Top 15	Top 10	Top 5
BBS	0.828	0.856	0.866	0.785	0.793	0.782
Tug	0.899	0.913	0.921	0.881	0.875	0.866
TUG + BBS	0.883	0.910	0.922	0.799	0.812	0.860

**Table 7:** Comparing the effect of including or excluding MSE features on the average precision and average recall values for the relevant clinical scores.

		With MSE Features			Without MSE Features		
Clinical Test		Top 15	Top 10	Top 5	Top 15	Top 10	Top 5
BBS	Precision	0.829	0.855	0.83	0.7562	0.7875	0.809
	Recall	0.841	0.862	0.846	0.816	0.832	0.826
TUG	Precision	0.869	0.861	0.866	0.841	0.848	0.861
	Recall	0.863	0.861	0.861	0.842	0.845	0.861
BBS + TUG	Precision	0.843	0.88	0.913	0.814	0.855	0.87
	Recall	0.869	0.899	0.915	0.864	0.877	0.891

**Table 8:** Results for the Kolgomorov–Smirnov test for normality.

	With MSE						Without MSE					
Clinical Test	Top 15	Top 10	Top 5	BBS	TUG	BBS + TUG	Top 15	Top 10	Top 5	BBS	TUG	BBS + TUG
KS	0.332	0.409	0.421	0.312	0.255	0.297	0.370	0.332	0.401	0.331	0.234	0.338
*p*-value	0.782	0.571	0.537	0.852	0.967	0.893	0.677	0.783	0.591	0.787	0.985	0.764

**Table 9:** *t*-test *p*-values for the comparison of including or excluding MSE from the feature set.

	Top 15	Top 10	Top 5	BBS	TUG	TUG + BBS
*t*-Test	0.064	0.031	0.008	0.017	0.037	0.008

## Data Availability

We have signed contracts with the hospital which prevents us from distributing or uploading the data collected.

## References

[1] Kamińska M.S., Brodowski J., Karakiewicz B. (2015). Fall Risk Factors in Community-Dwelling Elderly Depending on Their Physical Function, Cognitive Status and Symptoms of Depression. Int. J. Environ. Res. Public Health.

[2] Bergland A. (2012). Fall risk factors in community-dwelling elderly people. Nor. Epidemiol..

[3] Chu L.W., Chi I., Chiu A.Y.Y. (2005). Incidence and predictors of falls in the Chinese elderly. Ann. Acad. Med..

[4] Stevens J., Corso P.S., Finkelstein E., Miller T. (2006). The costs of fatal and non-fatal falls among older adults. Inj. Prev..

[5] Litwin H., Erlich B., Dunsky A. (2017). The Complex Association between Fear of Falling and Mobility Limitation in Relation to Late-Life Falls: A SHARE-Based Analysis. J. Aging Health.

[6] Letts L., Moreland J., Richardson J.A., Coman L., Edwards M., Ginis K.M., Wilkins S., Wishart L. (2010). The physical environment as a fall risk factor in older adults: Systematic review and meta-analysis of cross-sectional and cohort studies. Aust. Occup. Ther. J..

[7] Feldman F., Chaudhury H. (2008). Falls and the Physical Environment: A Review and a New Multifactorial Falls-Risk Conceptual Framework. Can. J. Occup. Ther..

[8] Nelson R.C., Murlidhar A.A. (1990). Falls in the Elderly. Emerg. Med. Clin. N. Am..

[9] Tinetti M., Speechley M., Ginter S. (1988). Risk factors for falls among elderly persons living in the community. N. Engl. J. Med..

[10] Duncan P.W., Studenski S., Chandler J., Prescott B. (1992). Functional Reach: Predictive Validity in a Sample of Elderly Male Veterans. J. Gerontol..

[11] Woollacott M.H., Shumway-Cook A. (1990). Changes in Posture Control across the Life Span—A Systems Approach. Phys. Ther..

[12] Maki B.E., Holliday P.J., Topper A.K. (1991). Fear of Falling and Postural Performance in the Elderly. J. Gerontol..

[13] Mänty M., Heinonen A., Viljanen A., Pajala S., Koskenvuo M., Kaprio J., Rantanen T. (2009). Self-reported preclinical mobility limitation and fall history as predictors of future falls in older women: Prospective cohort study. Osteoporos. Int..

[14] Rubenstein L.Z. (2006). Falls in older people: Epidemiology, risk factors and strategies for prevention. Age Ageing.

[15] Stubbs B., Schofield P., Binnekade T., Patchay S., Sepehry A., Eggermont L. (2014). Pain Is Associated with Recurrent Falls in Community-Dwelling Older Adults: Evidence from a Systematic Review and Meta-Analysis. Pain Med..

[16] Shumway-Cook A., Ciol M., Hoffman J., Dudgeon B.J., Yorkston K., Chan L. (2009). Falls in the Medicare Population: Incidence, Associated Factors, and Impact on Health Care. Phys. Ther..

[17] Luo Z.Q. (2012). Design and Development of a Portable Fall Risk Assessment System. Master’s Thesis.

[18] Montesinos L., Castaldo R., Pecchia L. (2018). Wearable Inertial Sensors for Fall Risk Assessment and Prediction in Older Adults: A Systematic Review and Meta-Analysis. IEEE Trans. Neural Syst. Rehabil. Eng..

[19] Saber-Sheikh K., Bryant E.C., Glazzard C., Hamel A., Lee R.Y. (2010). Feasibility of using inertial sensors to assess human movement. Man. Ther..

[20] Patel M., Pavic A., Goodwin V.A. (2019). Wearable inertial sensors to measure gait and posture characteristic differences in older adult fallers and non-fallers: A scoping review. Gait Posture.

[21] Wang K., Delbaere K., Brodie A.M.D., Lovell N., Kark L., Lord S.R., Redmond S.J. (2017). Differences Between Gait on Stairs and Flat Surfaces in Relation to Fall Risk and Future Falls. IEEE J. Biomed. Health Inform..

[22] Ponti M., Bet P., Oliveira C.L., Castro P.C. (2017). Better than counting seconds: Identifying fallers among healthy elderly using fusion of accelerometer features and dual-task Timed up and go. PLoS ONE.

[23] Howcroft J., Lemaire E.D., Kofman J., McIlroy W.E. (2018). Dual-Task Elderly Gait of Prospective Fallers and Non-Fallers: A Wearable-Sensor Based Analysis. Sensors.

[24] Hsu Y.C., Zhao Y., Huang K.-H., Wu Y.-T., Cabrera J., Sun T.-L., Tsui K.-L. (2020). A Novel Approach for Fall Risk Prediction Using the Inertial Sensor Data from the Timed-Up-and-Go Test in a Community Setting. IEEE Sens. J..

[25] Viton F., Elbattah M., Guerin J.-L., Dequen G. Heatmaps for Visual Explainability of CNN-Based Predictions for Multivariate Time Series with Application to Healthcare. Proceedings of the 2020 IEEE International Conference on Healthcare Informatics (ICHI).

[26] Hsieh T.Y., Wang S., Sun Y., Honavar V. Explainable Multivariate Time Series Classification: A Deep Neural Network Which Learns to Attend to Important Variables as well as Time Intervals. Proceedings of the 14th ACM International Conference on Web Search and Data Mining, Virtual.

[27] Aicha A.N., Englebienne G., Van Schooten K.S., Pijnappels M., Kröse B. (2018). Deep Learning to Predict Falls in Older Adults Based on Daily-Life Trunk Accelerometry. Sensors.

[28] Lee Y., Yoon S., Kim W. A Study on CNN-Based Berg Balance Scale Analysis for Elderly Persons. Proceedings of the 2019 34th International Technical Conference on Circuits/Systems, Computers and Communications (ITC-CSCC).

[29] Tunca C., Salur G., Ersoy C. (2019). Deep Learning for Fall Risk Assessment with Inertial Sensors: Utilizing Domain Knowledge in Spatio-Temporal Gait Parameters. IEEE J. Biomed. Health Inform..

[30] Kiprijanovska I., Gjoreski H., Gams M. (2020). Detection of Gait Abnormalities for Fall Risk Assessment Using Wrist-Worn Inertial Sensors and Deep Learning. Sensors.

[31] Aziz W., Arif M. Multiscale Permutation Entropy of Physiological Time Series. Proceedings of the 2005 Pakistan Section Multitopic Conference.

[32] Gruber A.H., Busa M.A., Iii G.E.G., Van Emmerik R.E., Masso P.D., Hamill J. (2011). Time-to-contact and multiscale entropy identify differences in postural control in adolescent idiopathic scoliosis. Gait Posture.

[33] Costa M., Peng C.-K., Goldberger A.L., Hausdorff J.M. (2003). Multiscale entropy analysis of human gait dynamics. Phys. A Stat. Mech. Appl..

[34] Riva F., Toebes M., Pijnappels M., Stagni R., van Dieën J. (2013). Estimating fall risk with inertial sensors using gait stability measures that do not require step detection. Gait Posture.

[35] Lee C.-H., Sun T.-L., Jiang B.C., Choi V.H. (2016). Using Wearable Accelerometers in a Community Service Context to Categorize Falling Behavior. Entropy.

[36] Bandt C., Pompe B. (2002). Permutation Entropy: A Natural Complexity Measure for Time Series. Phys. Rev. Lett..

[37] Lee C.-H., Chen S.-H., Jiang B.C., Sun T.-L. (2020). Estimating Postural Stability Using Improved Permutation Entropy via TUG Accelerometer Data for Community-Dwelling Elderly People. Entropy.

[38] Palumbo P., Palmerini L., Bandinelli S., Chiari L. (2015). Fall Risk Assessment Tools for Elderly Living in the Community: Can We Do Better?. PLoS ONE.

[39] Cella A., De Luca A., Squeri V., Parodi S., Vallone F., Giorgeschi A., Senesi B., Zigoura E., Guerrero K.L.Q., Siri G. (2020). Development and validation of a robotic multifactorial fall-risk predictive model: A one-year prospective study in community-dwelling older adults. PLoS ONE.

[40] Podsiadlo S., Richardson D. (1991). The Timed Up and Go: A Test of Basic Functional Mobility for Frail Elderly Persons. J. Am. Geriatr. Soc..

[41] McMurdo M.E.T. (2002). Guideline for the prevention of falls in older persons: Essential reading. Age Ageing.

[42] Salarian A., Horak F.B., Zampieri C., Carlson-Kuhta P., Nutt J.G., Aminian K. (2010). iTUG, a Sensitive and Reliable Measure of Mobility. IEEE Trans. Neural Syst. Rehabil. Eng..

[43] Greene B.R., Doheny E.P., O’Halloran A., Kenny R.A. (2013). Frailty status can be accurately assessed using inertial sensors and the TUG test. Age Ageing.

[44] Alexandre T.S., Meira D.M., Rico N.C., Mizuta S.K. (2012). Accuracy of Timed Up and Go Test for screening risk of falls among community-dwelling elderly. Braz. J. Phys. Ther..

[45] Chou C., Chien C., Hsueh I., Sheu C., Wang C., Hsieh C. (2006). Developing a Short Form of the Berg. Phys. Ther..

[46] Berg K., Wood-Dauphinee S., Williams J.I. (1995). The balance scale: Reliability assessment with elderly residents and patients with an acute stroke. Scand. J. Rehabil. Med..

[47] Karthikeyan G., Sheikh S.G., Chippala P. (2012). Test-retest reliability of short form of berg balance scale in elderly people. J. Med. Med. Sci..

[48] Shahzad A., Ko S., Lee S., Lee J.-A., Kim K. (2017). Quantitative Assessment of Balance Impairment for Fall-Risk Estimation Using Wearable Triaxial Accelerometer. IEEE Sens. J..

[49] Pfeiffer E. (1975). A Short Portable Mental Status Questionnaire for the Assessment of Organic Brain Deficit in Elderly Patients†. J. Am. Geriatr. Soc..

[50] Erkinjuntti T., Sulkava R., Wikström J., Autio L. (1987). Short Portable Mental Status Questionnaire as a Screening Test for Dementia and Delirium among the Elderly. J. Am. Geriatr. Soc..

[51] Tinetti M.E., Inouye S.K., Gill T.M., Doucette J.T. (1995). Shared Risk Factors for Falls, Incontinence, and Functional Dependence. J. Am. Med. Assoc..

[52] Van Doorn C., Gruber-Baldini A.L., Zimmerman S., Hebel J.R., Port C.L., Baumgarten M., Quinn C.C., Taler G., May C., Magaziner J. (2003). Dementia as a Risk Factor for Falls and Fall Injuries Among Nursing Home Residents. J. Am. Geriatr. Soc..

[53] Oliver D., Connelly J.B., Victor C., Shaw F.E., Whitehead A., Genc Y., Vanoli A., Martin F.C., Gosney M.A. (2006). Strategies to prevent falls and fractures in hospitals and care homes and effect of cognitive impairment: Systematic review and meta-analyses. Br. Med. J..

[54] Mecocci P., von Strauss E., Cherubini A., Ercolani S., Mariani E., Senin U., Winblad B., Fratiglioni L. (2005). Cognitive Impairment Is the Major Risk Factor for Development of Geriatric Syndromes during Hospitalization: Results from the GIFA Study. Dement. Geriatr. Cogn. Disord..

[55] Anstey K.J., Von Sanden C., Luszcz M.A. (2006). An 8-Year Prospective Study of the Relationship between Cognitive Performance and Falling in Very Old Adults. J. Am. Geriatr. Soc..

[56] Lin C.-H., Liao K.-C., Pu S.-J., Chen Y.-C., Liu M.-S. (2011). Associated Factors for Falls among the Community-Dwelling Older People Assessed by Annual Geriatric Health Examinations. PLoS ONE.

[57] Granger C.V., Dewis L.S., Peters N.C., Sherwood C.C., Barrett J.E. (1979). Stroke rehabilitation: Analysis of repeated Barthel index measures. Arch. Phys. Med. Rehabil..

[58] Howcroft J., Kofman J., Lemaire E.D. (2013). Review of fall risk assessment in geriatric populations using inertial sensors. J. Neuroeng. Rehabil..

[59] Bet P., Castro P.C., Ponti M. (2019). Fall detection and fall risk assessment in older person using wearable sensors: A systematic review. Int. J. Med. Inform..

[60] Lee C.-H., Sun T.-L. (2018). Evaluation of postural stability based on a force plate and inertial sensor during static balance measurements. J. Physiol. Anthr..

[61] Costa M., Goldberger A.L., Peng C.-K. (2002). Multiscale Entropy Analysis of Complex Physiologic Time Series. Phys. Rev. Lett..

[62] Richman J.S., Moorman J.R. (2000). Physiological time-series analysis using approximate entropy and sample entropy maturity in premature infants Physiological time-series analysis using approximate entropy and sample entropy. Am. J. Physiol. Hear. Circ. Physiol..

[63] Gu Y., Liang Z., Hagihira S. (2019). Use of Multiple EEG Features and Artificial Neural Network to Monitor the Depth of Anesthesia. Sensors.

[64] Breiman L. (2001). Random Forests. Mach. Learn..

[65] Niu D., Wang K., Sun L., Wu J., Xu X. (2020). Short-term photovoltaic power generation forecasting based on random forest feature selection and CEEMD: A case study. Appl. Soft Comput..

[66] Li X., Chen W., Zhang Q., Wu L. (2020). Building Auto-Encoder Intrusion Detection System based on random forest feature selection. Comput. Secur..

[67] Zhong Y., He J., Chalise P. (2020). Nested and repeated cross validation for classification model with high-dimensional data. Rev. Colomb. Estad..

[68] Daines K.J.F., Baddour N., Burger H., Bavec A., Lemaire E.D. (2021). Fall risk classification for people with lower extremity amputations using random forests and smartphone sensor features from a 6-minute walk test. PLoS ONE.

[69] Pedregosa F., Varoquaux G., Gramfort A., Michel V., Thirion B., Grisel O., Blondel M., Prettenhofer P., Weiss R., Dubourg V. (2011). Scikit-learn: Machine Learning in Python. J. Mach. Learn. Res..

[70] Riedl M., Müller A., Wessel N. (2013). Practical considerations of permutation entropy: A tutorial review. Eur. Phys. J. Spéc. Top..

